# HIV-1 interaction with an *O*-glycan-specific bacterial lectin enhances virus infectivity and resistance to neutralization by antibodies

**DOI:** 10.21203/rs.3.rs-2596269/v2

**Published:** 2024-01-03

**Authors:** Daniel W. Heindel, Dania M. Figueroa Acosta, Marisa Goff, Clauvis Kunkeng Yengo, Muzafar Jan, Xiaomei Liu, Xiao-Hong Wang, Mariya I. Petrova, Mo Zhang, Manish Sagar, Phillip Barnette, Shilpi Pandey, Ann J. Hessell, Kun-Wei Chan, Xiang-Peng Kong, Benjamin K. Chen, Lara K. Mahal, Barbara A. Bensing, Catarina E. Hioe

**Affiliations:** 1 Divison of Infectious Diseases, Department of Medicine, Icahn School of Medicine at Mount Sinai, New York, NY, USA; 2 Present address: Department of Biochemistry, Government Degree College Handwara, University of Kashmir, Jammu & Kashmir, India; 3 VA New York Harbor Healthcare System-Manhattan, New York, New York, United States of America.; 4 Department of Bioscience Engineering, University of Antwerp, Antwerp, Belgium; 5 Present address: Microbiome Insights and Probiotics Consultancy, Karlovo, Bulgaria; 6 Department of Medicine, Boston University Chobanian & Avedisian School of Medicine, Boston, MA, USA; 7 Department of Virology, Immunology and Microbiology, Boston University Chobanian & Avedisian School of Medicine, Boston, USA; 8 Division of Pathobiology and Immunology, Oregon National Primate Research Center, Oregon Health and Science University, Beaverton, OR, USA; 9 Department of Biochemistry and Molecular Pharmacology New York University Grossman School of Medicine, New York, NY, USA; 10 Department of Chemistry, University of Alberta, Edmonton, Alberta, Canada; 11 Department of Medicine, San Francisco Veterans Affairs Medical Center and University of California, San Francisco, CA, USA; 12 James J. Peters VA Medical Center, Bronx, USA

**Keywords:** HIV, bacteria, lectin, glycosylation, O-glycan

## Abstract

Bacteria dysbiosis has been associated with an increased risk of HIV-1 transmission and acquisition. The prevalent idea is that bacteria dysbiosis compromises mucosal integrity and promotes inflammatory conditions to cause recruitment and activation of immune cells that harbor or are targeted by HIV-1. However, it is also possible that HIV-1 directly binds bacteria or bacterial products to impact virus infectivity and transmissibility. This study evaluated HIV-1 interactions with bacteria through glycan-binding lectins. The *Streptococcal* Siglec-like lectin SLBR-N, which is part of the fimbriae shrouding the bacteria surface and recognizes α2,3 sialyated *O*-linked glycans, was noted for its ability to enhance HIV-1 infectivity in the context of cell-free infection and cell-to-cell transfer. Enhancing effects were recapitulated with *O*-glycan-binding plant lectins, signifying the importance of *O*-glycans. Conversely, *N*-glycan-binding bacterial lectins FimH and Msl had no effect. SLBR-N was demonstrated to capture and transfer infectious HIV-1 virions, bind to *O*-glycans on HIV-1 Env, and increase HIV-1 resistance to broadly neutralizing antibodies targeting different regions of Env. Hence, this study highlights the potential contribution of *O*-glycans in promoting HIV-1 infection through the exploitation of *O*-glycan-binding lectins from commensal bacteria at the mucosa.

## Introduction

HIV-1 transmission often occurs through mucosal surfaces during sexual contact or during the perinatal period, including pregnancy, childbirth and breastfeeding^1^. While bacterial dysbiosis at the mucosa and accompanying inflammation have been associated with an increased risk of HIV-1 transmission, little is known about whether direct interactions between HIV-1 and bacteria that are part of the human mucosal microbiome contribute to HIV-1 transmissibility. There are precedents for intimate connections between viruses and bacteria^2, 3^. Human norovirus, for example, requires a specific histo-blood group antigen (HBGA) expressed by the commensal bacteria *Enterobacter cloacae* to augment infection in B cells; these HBGA glycans bind directly to norovirus particles through the major capsid protein, but their roles in infection are poorly understood^3–5^. Other HBGA-expressing bacteria help protect norovirus from heat stress^6^. Poliovirus binds, via its VP1 capsid, to bacterial lipopolysaccharide (LPS) resulting in increased virus thermostability and resistance to inactivation by chlorine bleach, as well as enhanced binding to the cellular poliovirus receptor^7^. Reovirus also interacts directly with bacterial LPS and peptidoglycan, which augment virus thermostability and attachment to mammalian cells^8^. When mice are depleted of bacterial microbiota by antibiotic treatment before oral exposure to poliovirus or reovirus, they show decreased virus infection and pathology in the intestine^9^. While direct virus-bacteria interactions and their biological consequences have been well studied for these enteric and other viruses, such as murine mammary tumor virus^10, 11^, evidence to show whether HIV-1 can interact with bacteria or bacterial products to impact its infection and transmission is still lacking.

On the virion surface, HIV-1 displays membrane-anchored envelope (Env) glycoproteins with dense glycosylation that encloses most of the Env surface. Each of the three Env monomeric subunits contain both *N*- and *O*-glycans with heterogeneous compositions. As many as 30 *N*-glycans can be found per Env monomer^12^, masking this sole viral surface antigen from immune recognition^13–15^. Our understanding of *O*-glycans on Env is more limited. Initial reports concerning *O*-glycans on HIV-1 Env were discordant^16, 17^, however a later publication verified the discovery of *O*-glycans on HIV-1 Env gp120 from different isolates^18^. Specifically, out of the eight mammalian core *O*-glycan structures, core 1 and core 2 structures were identified in the V1 region by mass spectrometry (MS) and the number of predicted *O*-glycosylation sites correlated with the V1 length. An earlier MS analysis of virion-associated gp120 also detected a core 1 *O*-glycan at the highly conserved C5 site located upstream from the furin-cleavage site^19^. The presence of *O*-glycans is not limited to HIV-1, as core 1 and core 2 *O*-glycans have similarly been observed on SIV and HIV-2 gp120s^20^. In a cryo-EM structure of SIV Env in complex with broadly neutralizing antibody (bNAb) PGT145 and *O*-glycan-specific lectin jacalin (AIA), jacalin was found to bind to a V1 *O*-glycan^21^. Notably, the presence of *O*-glycans on V1 were found to reduce virus recognition and neutralization by bNAbs against the V3-glycan epitopes^18^, indicating a role for *O*-glycans, similar to *N*-glycans, in shielding antibody epitopes. Nonetheless, the functions of *O*-glycans on Env remain unclear.

Many studies have shown that *N*-linked glycans on HIV-1 Env interact with glycan-binding proteins (lectins) to impact virus infection and transmission. Algae or plant lectins specific for high mannose-type *N*-glycans, including griffithsin (GRFT), scytovirin (SVN), cyanovirin-N (CVN), and *Galanthus nivalis* agglutinin (GNA), have varying levels of inhibitory activity against HIV-1 isolates^22^. On the other hand, innate immune lectins on host cells that recognize the same or similar *N*-glycan structures can promote HIV-1 infection. For example, DC-SIGN binds high mannose *N*-glycans and is expressed on the surface of certain types of dendritic cells and macrophages at mucosal sites. DC-SIGN can function as a receptor to capture HIV-1 virions and mediate in trans-infection by presenting captured virions to permissive CD4 T cells^23,24, 25^. Siglec-1 is expressed on more mature dendritic cells and also participates in HIV-1 trans-infection through recognition of α−2,3 sialic acid on HIV-1^26^. Siglec-7, a cellular lectin with specificity for terminal α(2,8) or α(2,6) sialic acids^27^, binds HIV-1 gp120, and its soluble form has been found to facilitate HIV-1 entry to CD4+ T-cells and macrophages^28^. The mechanism by which soluble Siglec-7 affects virus entry is yet unknown, but it does not facilitate virus adherence to target cells as the membrane-bound Siglec-7 or Siglec-1 do^28–30^. Lectins are also expressed by different bacteria species at the tips of their fimbriae for adherence to host cell-surface glycans and colonization^31^; however, the interactions between such bacterial lectins and HIV-1 glycans and their consequences on HIV-1 infectivity have not been explored.

In this study, we investigated the interaction between HIV-1 and a panel of bacterial lectins and evaluated their effects on virus infectivity and neutralization by antibodies. These lectins are specific for high-mannose *N*-glycans or sialyated *O*-glycans, which were among the glycan types enriched in HIV-1 virions. They are expressed at the tips of fimbriae that protrude from the bacterial cell wall and shroud the bacteria surface, and can be shed into the milieu. In particular we tested lectins from bacteria that are part of the human mucosal microbiota: FimH, a lectin of uropathogenic *Escherichia coli* with high affinity for *N*-linked high mannose Man_5_^32, 33^; Msl from vaginal non-pathogenic *Lactiplantibacillus plantarum* CMPG5300 that binds high-mannose Man_5–9_ *N*-glycans^34^; SLBR lectins (SLBR-B, SLBR-H, and SLBR-N) from oral nonpathogenic *Streptococcus gordonii strains* that bind sialylated *O*-glycans^35^. This study revealed the ability of these bacterial lectins to bind HIV-1 Env and elicit different effects on virus infectivity. Notably, the *O*-glycan-specific lectin SLBR-N was found to enhance HIV-1 infection in the context of both cell-free virus infection and a CD4-dependent cell-to-cell viral transfer assay while also rendering the virus more resistant to neutralizing antibodies. This enhancement was recapitulated to varying extents by other lectins recognizing *O*-glycans and not by the *N*-glycan-binding lectins, signifying the critical involvement of *O*-glycan engagement. Hence, this is the first study to demonstrate a direct interaction between HIV-1 and bacteria through fimbrial lectins and to highlight an important novel role for *O*-glycans in influencing HIV-1 infectivity and transmissibility.

## Results

### Enrichment of high-mannose *N*-glycans and core 1 and core 3 *O*-glycans in HIV-1 virions as detected by lectin microarray analysis.

To understand the biological importance of glycans present on HIV-1 virions, we determined their glycomic signature using a dual-color lectin microarray technology^36, 37^ (**Supplemental Table 1**). Four virus strains of different clades were analyzed. The data in **Supplemental Figure 1A** revealed 35 lectins out of 116 tested with higher binding to virus over cell lysates, and of these, three sets of lectins with distinct glycan specificities are highlighted herein.

The first set is composed of lectins specific for mannose terminated *N*-glycans. Consistent with well documented findings from our lab and others showing mannose glycans on HIV-1 Env^22, 23, 38–40^, we observed increased binding for three mannose-specific lectins (GNA Sigma, GNA Vector, and Con A) to virus versus cell lysates (**Supplemental Figure 1A, 1B left panel**). Secondly, we detected enhanced binding for MAA lectins, which recognize α2,3 sialic acid or sulfation present on either *N*- or *O*-glycans (**Supplemental Figure 1A and 1B middle panel**). The third set of lectins with higher binding to virus lysates were AIA GlycoMatrix, AIA Vector, and MPL Vector, all of which are specific for core 1 and core 3 *O*-glycans (**Supplemental Figure 1A and Supplemental Figure 1B right panel**). Multiple *O*-glycosylation sites were predicted to be present on each of the HIV-1 Env strains examined in this and subsequent experiments: many were located in the V1V2, V4, and V5 loops, one in a conserved C5 site upstream from the furin gp120-gp41 cleavage site, and few in the extracellular gp41 region (**Supplemental Figure 2**). These data are in line with past studies that experimentally identified *O*-glycans on various Envs^18, 21^ and with previous lectin microarray analysis of HIV-1 virions^37^. Altogether, the data demonstrate an accumulation of high mannose *N*-glycans and core 1 and/or core 3 *O*-glycans on HIV-1 virions relative to the cells producing these viruses.

### Enhanced HIV-1 infectivity upon treatment with bacterial lectins that bind *O*-glycans but not with lectins specific for high mannose *N*-glycans.

We next examined the effects of lectins binding to high-mannose *N*-glycans or *O*-glycans on HIV-1 infection. We focused on lectins expressed by bacteria species that are part of the human mucosal microbiota: high-mannose N-glycan-binding FimH from uropathogenic *E. coli*, high-mannose N-glycan-specific Msl from vaginal *L. plantarum*, and *O*-glycan-specific SLBR lectins (SLBR-B, SLBR-H, and SLBR-N) from oral commensal *Streptococcus gordonii* strains. The binding specificities of these lectins are shown in **Table 1**. High mannose-specific lectin GRFT, known to have potent anti-HIV-1 activity ^22, 23, 41^, was included as a control. *O*-glycan-binding plant lectins (AIA, MAL II) were also examined for comparison. Titrated amounts of each lectin were pre-incubated with virus for 1 hour at 37°C. HIV-1 Infectious molecular clones (IMCs) of acute or transmitted/founder isolates from subtypes C and CRF_01.AE (Z331M and CMU06) were tested. The mixtures were then added to TZM.bl reporter cells and virus infection was measured after 48 hours.

**Figure 1:**
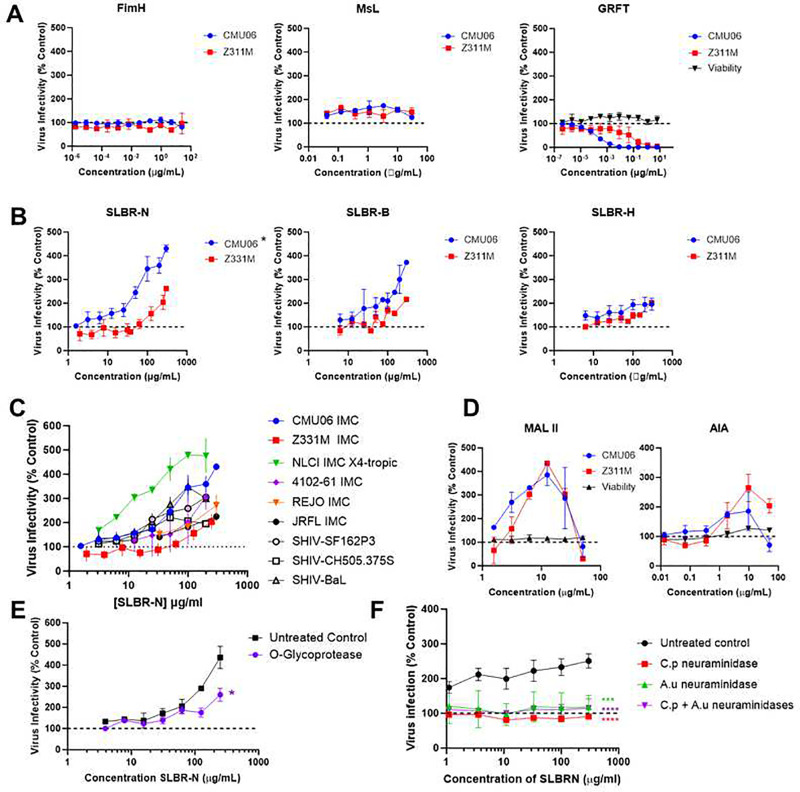
Differential impact of bacterial and plant lectins on HIV-1 infectivity. (A-B) HIV-1 virions were pre-incubated with titrated amounts of bacterial lectins for 1 hour at 37° C and assessed for infectivity in TZM.bl reporter cells after 48 hours. Untreated virions served as control (100% infectivity; dotted lines). Full length infectious molecular clones of HIV-1 tested included CRF_01.AE CMU06 and clade C Z331M. Bacterial lectins specific for high-mannose (A) or α2,3 sialyated *O*-linked glycans (B) were tested. Cell viability was measured using PrestoBlue HS Cell Viability reagent (Invitrogen). * denotes p=0.0008, p=0.0033, and p=0.17 for CMU06 treatment with SLBR-N vs. SLBR-H, SLBR-N vs. SLBR-B, and SLBR-H vs. SLBR-B, respectively, by two way ANOVA. (C) Effects of SLBR-N treatment were examined as in panel B on additional HIV-1 and SHIV strains. (D) Effects of plant lectins specific for *O*-linked cr2,3 sialylated glycans (MALII) and core 1 or core 3 *O*-glycans (AIA) were also examined for comparison. Cell viability was measured as above. (E-F) To validate the presence of *O*-glycans and verify that the enhancing effect of *O*-glycan-specific lectins was glycan dependent, virus was pretreated with *O*-glycoprotease (E) or neuraminidases from *Clostridium perfringens* (C.p) or *Arthrobacter ureafaciens* (A.u) or both (F). *, p<0.05, ***, p<0.001, ****, p<0.0001 by two-way ANOVA vs. untreated control Each experiment was performed at least twice. Mean and standard error from repeat experiments are shown.

The data demonstrate that treatment with FimH or Msl neither inhibited nor enhanced viral infection in a dose-dependent manner, whereas as expected, GRFT inhibited both tested viruses with different potencies (Figure 1A). Surprisingly, virus treatment with SLBR-N enhanced virus infection in a dose-dependent manner, and the enhancement levels varied for the different virus strains (Figure 1B left panel). In contrast, the other two SLBR lectins had more modest effects (Figure 1B middle and right panels). Because of the greater potency of SLBR-N, this lectin was selected for further testing in subsequent experiments. SLBR-N was tested on additional virus strains and the varying levels of enhanced infection were similarly observed (Figure 1C). Enhanced infection also was observed upon virus treatment with two *O*-glycan-specific plant lectins (MAL II and AIA) although the enhancing effects declined at higher concentrations (Figure 1D), suggesting a distinct mechanism. Enhanced infection by *O*-glycan specific lectins (SLBR-N, AIA, and MAL II) was maintained whether the viruses were produced in 293T cells or PBMCs (**Supplemental Figure 3A**). Enhancement was also seen in the presence or absence of a GST tag on SLBR-N (**Supplemental Figure 3B**). The presence of *O*-glycans on HIV-1 was further verified by an observed reduction of virus infectivity upon virus treatment with O-glycoprotease, an enzyme that cleaves the peptide bond N-terminal to a Ser or Thr containing an *O*-glycan (Figure 1E). To validate that lectin-glycan interactions are responsible for the observed increase of virus infectivity, we pretreated virus with neuraminidases that remove terminal α−2,3 sialic acids critical for SLBR-N. The sialic acid removal abrogated the capacity of SLBR-N to enhance virus infectivity (Figure 1F). Virus treated only with O-glycoprotease remained infectious but showed reduced infectivity, whereas neuraminidase treatment increased virus infectivity to some extent as previously shown^42, 43^ (**Supplemental Figure 4A-B**). We also assessed the glycan dependence of AIA activity by pretreating AIA with melibiose, a soluble disaccharide recognized by AIA^44^. Treatment tempered the enhancement of virus infectivity by AIA, suggesting that AIA-glycan interactions are responsible (**Supplemental Figure 4C**). These data suggest that the engagement of *O*-glycans on HIV-1 virions by different lectins, including bacterial SLBR-N from oral-colonizing *Streptococcus gordinii*, resulted in increased virus infectivity, while the interaction with high-mannose N-glycan-binding bacterial lectins FimH and Msl did not affect infectivity.

### Bacterial lectins bind HIV-1 virions and Env in a glycan-dependent manner.

**Figure 2:**
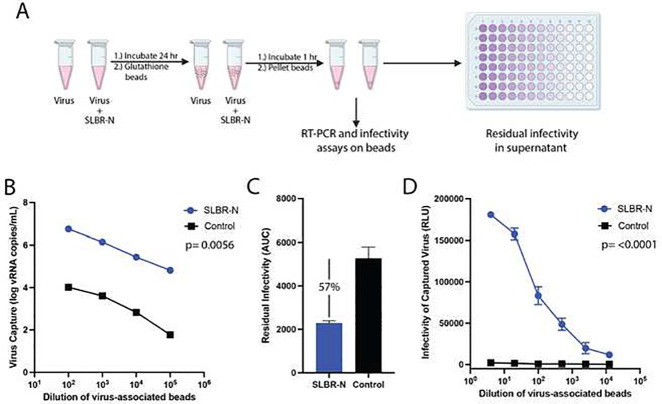
Capture of HIV-1 virions and recognition of gp120 by the SLBR-N lectin. (A) Schematic of the experimental procedure. Virus was incubated with GST-tagged SLBR-N for 24 hours at 37° C and then glutathione beads were added. After the beads were pelleted, qRT-PCR or infectivity assays were performed on the beads while the supernatant was titrated on TZM.bl cells to measure residual infectivity. (B) The amount of CMU06 virus captured by SLBR-N-coated beads or control beads as measured by qRT-PCR. (C) The residual infectivity of CMU06 in the supernatant after capture with SLRB-N-coated beads versus control beads was calculated as areas under the titration curve (AUC). Error is shown as SD for 3 technical replicates. The percentage of HIV-1 captured by SLBR-N vs control is shown above the bar graph. (D) The infectivity of JRFL virus captured by SLBR-N coated beads or control beads upon incubation with TZM.bl cells as measured by luminescence (RLU). A two tailed t test was used in panel B and a two-way ANOVA in panel D to compare SLBR-N treated vs control. Data from one set of repeat experiments are shown.

We next tested whether bacterial lectins can engage HIV-1 virions via viral glycans using a virus capture assay outlined in Figure 2A. Virus was incubated with GST-tagged SLBR-N for 24 hours at 37°C. The mixture was then incubated with glutathione beads and the virion bound beads were pelleted. The beads were washed to remove unbound virions and subjected to qRT-PCR and infectivity assays, while the supernatant was titrated on TZM.bl cells to measure residual virus infectivity. The input virus was also treated with beads in the absence of SLBR-N and analyzed in parallel to serve as a control.

We detected higher levels of viral RNA for beads with SLBR-N-treated CMU06 versus control as measured by qRT-PCR (Figure 2B), indicating the capture of HIV-1 particles by SLBR-N. Conversely, the residual infectivity of the corresponding supernatant was reduced compared to the control (Figure 2C). These experiments were performed with a transmitted/founder isolate Z331M and comparable results were observed (**Supplemental Figure 5A, B**). Importantly, the virions captured on SLBR-N-coated beads maintained infectivity (Figure 2D). For comparison, we also examined FimH which displayed no effect on virus infectivity (Figure 1A) and found that FimH captured both CMU06 and Z331M (**Supplemental Figure 5C**). These data show that both bacterial lectins α−2–3 sialylated *O*-glycan-specific SLBR-N and high mannose *N*-glycan-specific FimH interacted with viral surface glycans while having differential effects on virus infectivity.

**Figure 3:**
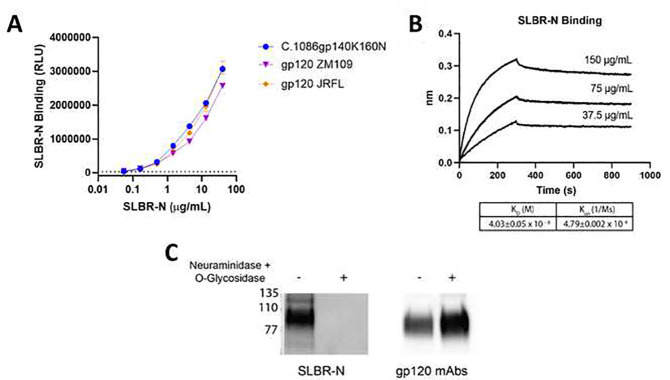
SLBR-N interaction with HIV-1 Env (A) SLBR-N binding to HIV-1 Env proteins of different virus strains was measured by E LISA. Recombinant soluble Env proteins were coated onto well surface and then treated with titrating amounts of GST-tagged SLBR-N. SLBR-N binding was detected by rabbit anti-GST antibody followed by HRP-conjugated anti-rabbit IgG. Background binding (SLBR-N binding to no Env) is indicated by dotted line. (B) The binding affinity of SLBR-N for HIV-1 gp120 ZM109 was determined using Octet BLI. Recombinant gp120 was coupled to AR2G biosensors and then reacted with SLBR-N at the designated concentrations. (C) Recombinant ZM109 gp120 protein was subjected to SDS-PAGE, transferred to a nitrocellulose membrane and probed with SLBR-N lectin or an anti-gp120 mAb pool. The gp120 protein was treated with a mixture of neuraminidase and *O*-glycosidase to remove sialic acids and *O*-glycans. Loss of SLBR-N reactivity verified the *O*-glycan-dependent interaction between SLBR-N and HIV-1 Env. Experiments were repeated two or more times. Data from one of the repeat experiment are shown.

We next examined whether SLBR-N interacted with HIV-1 via the virus Env glycoprotein and determined if this interaction was glycan dependent. An ELISA was performed where recombinant gp120 or gp140 proteins coated on the plate were reacted with SLBR-N. We observed that SLBR-N recognized each of the three tested Env proteins in a dose-dependent manner (Figure 3A). We further characterized this interaction by determining the binding kinetics of SLBR-N for gp120 using Octet BLI (Figure 3B). SLBR-N interacted with gp120 ZM109 coupled to the AR2G biosensor in a concentration-dependent manner with a measured K_D_ of 40.3 nM. However, although gp120 ZM190 has a single predicted *O*-glycan site (**Supplemental Figure 2**), a binding stoichiometry greater than 1:1 could not be ruled out. The non-saturating association and incomplete dissociation phases also indicated heterogeneous multivalent interactions potentially due to some degree of SLBR-N oligomerization. We then used lectin blotting to show that the interaction between HIV-1 Env and SLBR-N was glycan dependent. gp120 ZM109 was untreated or pretreated with a mixture of neuraminidase and O-glycosidase to remove sialic acids and *O*-glycans and then probed with SLBR-N. The blots were subsequently stripped and re-probed with an anti-gp120 mAb pool to verify Env bands. SLBR-N recognized untreated gp120, and its reactivity was depleted upon enzyme treatment (Figure 3C), indicating the interaction of SLBR-N with HIV-1 Env depends on the presence of sialylated *O*-glycans. These data together indicate that SLBR-N recognizes α−2,3 sialylated *O*-glycans on HIV-1 Env.

For comparison, we also subjected recombinant gp120 proteins (LAI, IIIB, ZM109) to lectin blot and BLI analyses with FimH. FimH recognized different gp120 proteins to varying degrees (**Supplemental Figure 6A**), consistent with past data showing the heterogenous presence of high-mannose N-glycans on HIV-1 gp120 proteins^22, 23^. Treatment of gp120 ZM109 with EndoH or PNGaseF enzymes that removed high mannose or all *N*-glycans, respectively, abrogated FimH recognition (**Supplemental Figure 6B**), indicating that high mannose N-glycans on Env are required for FimH-Env interaction. The blots were re-probed with an anti-gp120 mAb pool to define the Env bands and verify the enzymatic glycan cleavage. FimH was also serially titrated and analyzed for its binding affinity to gp120 ZM109 by Octet BLI (**Supplemental Figure 6C**). We measured a K_D_ of 360 nM, in line with a past report of FimH affinity for Man_7_ glycans^33^. These data demonstrate that both SLBR-N and FimH interacted with HIV-1 virions and the virus Env in particular, while having distinct impacts on virus infectivity. SLBR-N binding resulted in enhanced virus infection, whereas upon FimH binding virus infectivity was unchanged.

### Enhanced virus infection upon SLBR-N treatment of HIV-1 virions and not target cells.

**Figure 4:**
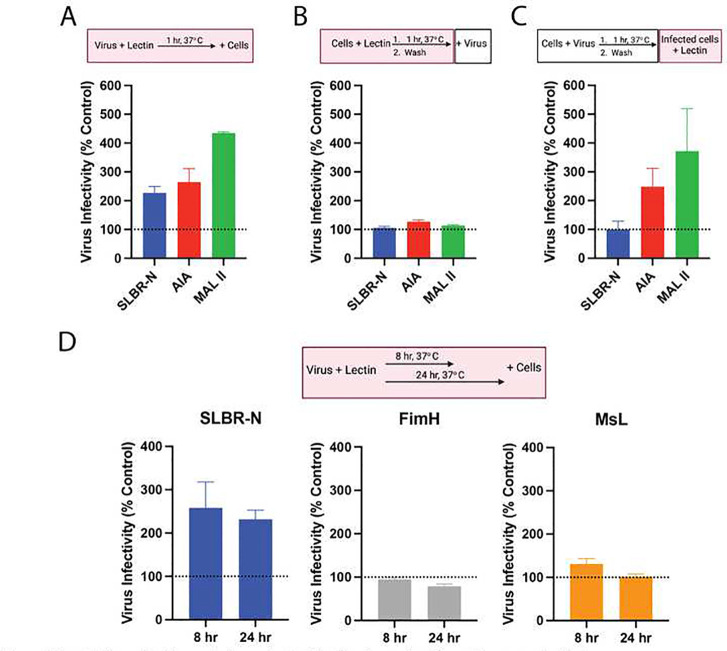
Incubation of lectins and virus prior to infection is required for enhancement effect. (A) Enhanced infection was observed when HIV-1 Z331M was pretreated with SLBR-N (100 μg/mL), AIA (12.5 μg/mL) and MAL II (12.5 μg/mL) for 1 hour at 37° C and then added to TZM.bl cells. (B) No enhancement was observed when TZM.bl cells were pretreated with SLBR-N, AIA, and MAL II at the aforementioned concentrations for 1 hour at 37°C, washed, and then infected with HIV-1 Z331M. (C) Enhanced infection was observed with AIA and MAL II but not SLBR-N when TZM.bl cells were first infected with Z331M virus, washed, and then treated with the lectins at the previously mentioned concentrations. (D) Enhanced infection was stably detected upon prolonged pretreatment of HIV-1 Z331M with SLBR-N (100 μg/mL) for 8 or 24 hours at 37°C, but not with FimH (25 μg/mL) and MsL (25 μg/mL). For all the above experiments, the relative levels of virus infection in the TZM.bl cells were measured at 48 hours post-infection by luminescence intensity. Virus infectivity was normalized to the untreated control for each experimental condition (set to 100%). Boxes above the graphs show the experimental outlines with red boxes highlighting the presence of lectin in the experiments. Mean and standard error from two repeat experiments are shown.

To better understand the mechanism by which the *O*-glycan-binding lectins SLBR-N, AIA, and MAL II augment HIV-1 infectivity, lectin treatment was applied at different time points during the infection assay as depicted in Figure 4. Each lectin was tested at a concentration that showed an enhancing activity in Figure 1B: SLBR-N (100 μg/mL), AIA (12.5 μg/mL) and MAL II (12.5 μg/mL). When lectin was added to the virus first and then mixed with TZM.bl target cells, an enhancement of virus infection was observed with all three lectins as compared to the untreated control (Figure 4A). In contrast, treatment of target cells with each of the three lectins prior to infection resulted in no enhancement (Figure 4B), confirming the importance of lectin interactions with HIV-1 rather than target cells in promoting virus infectivity. The data also suggest a low likelihood for cross-linking between virus and target cell by these lectins. Interestingly, when the cells were infected with virus first and then treated with lectins, enhanced infection was observed upon treatment with the two plant lectins (AIA and MAL II), but not with bacterial SLBR-N (Figure 4C), indicating that different steps of virus infection are affected by the plant versus bacterial lectins. This could be due to a difference in the number of carbohydrate-binding sites or the specific *O*-glycan structures recognized by the two classes of lectins (**Table 1**). Taken together this time-course experiment shows that the bacterial lectin SLBN-N enhanced HIV-1 infectivity mainly by interacting with the virus prior to infection of target cells, whereas AIA and MAL II promoted infection also at a post-infection step.

Subsequent experiments were performed to investigate the impact that prolonged virion-lectin interactions have on virion infectivity. HIV-1 virions were subjected to a preincubation with bacterial lectins SLBR-N, FimH, or Msl for 8 or 24 hours at 37°C prior to addition of TZM.bl target cells. SLBR-N pretreatment for both 8 and 24 hours led to enhanced virus infection as compared to untreated control (Figure 4D), recapitulating the enhancement seen with 1 hour pretreatment (Figure 4A). However, high mannose-specific lectins FimH and Msl had no impact even with prolonged preincubation. These data suggest the specific contribution of *O*-glycan-specific bacterial lectin SLBR-N in promoting the stability and infectivity of HIV-1 virions.

### Enhanced transfer of HIV-1 virions from cell to cell by SLBR-N.

**Figure 5:**
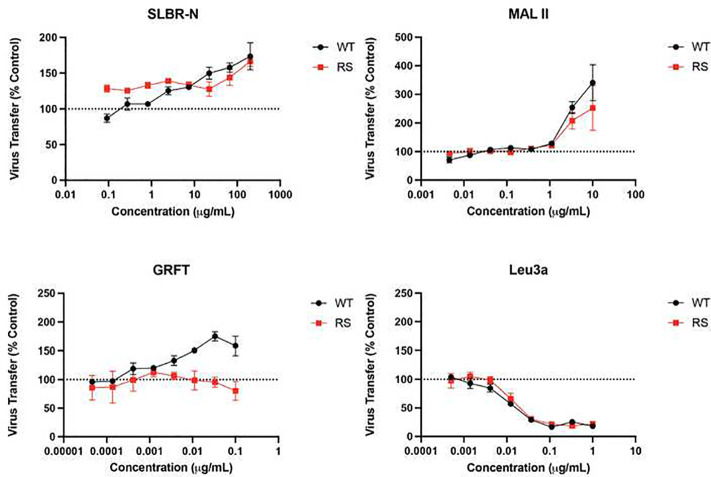
O-glycan-specific lectins enhance cell-cell transfer of HIV-1 at the pre-fusion step Jurkat cells nucleofected with an HIV-1 clone bearing T/F clade B EnvQH0692 and an mCherry reporter gene were used as donor cells, whereas primary CD4 T cells served as target cells. The two cell types were labeled with distinct dyes, mixed for 3 hours, and mCherry+ HIV-1 transfer to target cells was monitored by flow cytometry. Donor cells were pre-treated with titrating amounts of lectin before mixing with target cells. *O*-glycan-specific lectins SLBR-N and MAL II were tested in comparison with high mannose *N*-glycan-binding lectin GRFT. Anti-CD4 Leu3a mAb blocking gp120-CD4 engagement was tested as control. Viruses with WT Envor cleavage-defective RS Env (R508S/R511S) capable of binding CD4 but not virus fusion were tested in parallel. Error is calculated as SEM for two or three repeat experiments.

We further tested the effect of SLBR-N on cell-associated HIV-1 and the transfer of viral particles between cells, a highly efficient mode of HIV-1 spread in vitro^45, 46^. A 3-hour cell-to-cell virus transfer assay was performed using Jurkat T cells nucleofected with an HIV-1 clone bearing T/F Env B.QH0692 with a Gag-iCherry reporter as a donor cell. This clone produces intact virus particles that are highly fluorescent and allows viral particle transfer to be tracked by flow cytometry. The donor cells, which express HIV-1 Env at the cell surface, were then cocultured with primary CD4 T cells allowing for virological synapse formation upon HIV-1 Env/CD4 recognition, virus transfer to target cells, and internalization into a trypsin-resistant endocytic compartment, independent of virus fusion^45, 47, 48^. The two cell types were also labelled with distinct dyes (eFluor 450 and 660)^49^ to discriminate target cells from donors. Prior to co-culturing, donor cells were incubated with SLBR-N, MAL II, or GRFT. After 3 hour of co-incubation, mCherry+ virion transfer to target cells was monitored by flow cytometry (**Supplemental Figure 7**). Treatment of donor cells with SLBR-N increased mCherry+ WT virion transfer to CD4 T cells (Figure 5). Enhancement was similarly seen with *O*-glycan-specific MAL II. Virus transfer was blocked by the anti-CD4 mAb Leu3a included as a control, which indicates that the cell-to-cell transfer requires Env-CD4 engagement.

We then investigated the role of *O*-glycan-mediated enhancement in virus transfer using a gp120-gp41 cleavage-defective virus (due to R519S/R522S (RS) mutations at the REKR furin cleavage site)^50, 51^. Interestingly, *O*-glycan-binding lectins (SLBR-N and MAL II) demonstrated enhancement of the RS virus transfer, similar to that seen with WT (Figure 5). Because the RS mutant is capable of binding CD4 but not virus fusion, the results point to *O*-glycan-mediated enhancement at the initial step of virus-cell interaction prior to viral membrane fusion.

We noted a different pattern with GRFT. This high mannose-binding lectin augmented WT transfer (Figure 5), even though it had no effect on cell-free infection of 293T-derived viruses (Figure 1A). GRFT also did not affect the transfer of the RS virus (Figure 5). The data suggest differences in the high mannose glycan contents of Env expressed on Jurkat cells versus cell-free virions produced in 293T cells, and also on cleaved versus uncleaved Env proteins as we reported previously^40, 52^.

An ELISA was performed using recombinant C1086 gp140 K160N and CD4 proteins to detect if SLBR-N treatment of Env caused alterations to the CD4-gp120 interaction. The C1086 Env protein interacted in a dose-dependent manner with SLBR-N (Figure 3A) and bound CD4 with a half-maximal or EC50 value of 0.08 μg/ml (1.6 nM) (**Supplemental Figure 8**). We observed a negligible shift in the EC50 values for CD4 binding to SLBR-treated Env versus untreated Env (**Supplemental Figure 8**), indicating that SLBR-N has no direct effects on Env binding to its CD4 receptor and that other mechanisms are likely in play for this lectin to promote the initial virus-cell interactions.

### Reduced virus neutralization by bNAbs upon SLBR-N treatment.

**Figure 6.**
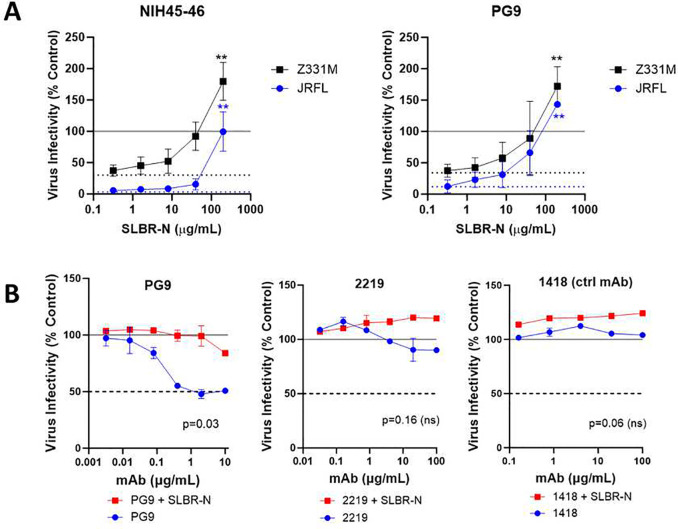
SLBR-N renders HIV-1 more resistant to neutralizing antibodies. A. HIV-1 (cladeB JRFL and cladeC Z331M) was treated with bnAbs against the CD4-bindingsite (NIH45–46) and the V1V2 glycan epitope (PG9) followed by increasing concentrations of SLBR-N. The bNAbs were used at a constant amount of 1 μg/mL (NIH45–46) and 10 μg/mL (PG9), which on their own inhibited virus by >50%. Blue and black dotted lines: respective levels of JRFL and Z331M neutralization by bNAbs alone. **, p<0.01 versus bNAb alone by Kurskal Wallis test. B. HIV-1 (JRFL) was treated with titrating amounts of the V1V2 glycan-specific bNAb PG9, a mAb against the cryptic V3 crown epitope (2219) or an irrelevant control mAb (1418) in the presence of SLBR-N (200 μg/mL) or no SLBR-N. Dotted lines denotes 50% neutralization, p values indicate significant (<0.05) or no significant (ns) difference between mAb neutralization in the presence and absence of SLBR-N (Wilcoxontest). Virus was treated with mAb plus or minus SLBR-N for 1 hour at 37°C and then incubated withTZM.bl target cells. After 48 hours, virus infection was measured by luciferase activity. Experiments were performed at least two times. Mean and standard error values from one of the experiments are shown.

Next, we wanted to test whether SLBR-N engagement affected virus neutralization by antibodies. HIV-1 JRFL and Z331M viruses were pretreated for 1 hour with bNAbs against the CD4-binding site (NIH45–46) or the V1V2 glycan epitope (PG9) at a concentration that achieved greater than 50% virus neutralization. The mixture was then added to titrated amounts of SLBR-N while keeping the bNAb concentration constant. After 48 hours, HIV-1 infectivity was measured by luciferase activity. We observed a reduction in the neutralizing capacity of both NIH45–46 and PG9 upon treatment with increasing amounts of SLBR-N from 0.3 to 200 μg/ml (Figure 6A). At >100 μg/ml SLBR-N, virus neutralization was significantly abrogated and enhanced virus infection was observed. PG9 was tested in another experimental condition, in which JRFL was treated with titrating amounts of this bNAb in the presence or absence of SLBR-N at a fixed concentration of 200 μg/ml (Figure 6B, left panel). JRFL neutralization by PG9 was significantly diminished in the presence of SLBR-N. These results demonstrate the ability of SLBR-N to overcome virus neutralization by bNAbs.

To examine if SLBR-N binding causes an extensive change to the Env conformation, neutralization was also examined with mAb 2219 against a cryptic V3 crown epitope, which is accessible only in an open Env conformation^53^. JRFL was treated with titrating amounts of mAb against a cryptic V3 crown (2219) plus or minus SLBR-N (200 μg/ml). As expected for a tier 2 virus with closed Env, JRFL was not neutralized by mAb 2219 (Figure 6B, middle panel). Upon SLBR-N treatment, the virus remained resistant to 2219. The same pattern was seen with an irrelevant mAb control 1418 (Figure 6B, right panel). Hence, while SLBR-N reduced the potency of CD4bs and V1V2 glycan bNAbs, it did not trigger an open Env conformation that would render the virus susceptible to mAb against the occluded V3 site. Nonetheless, there is still a possibility that SLBR-N binding to Env poses steric interference or induces allosteric changes on the Env regions that are targeted by the CD4bs and V1V2 glycan bNAbs.

## Discussion

This study provides evidence for direct interactions between HIV-1 and lectins from bacteria present in the host mucosal microbiota, and these interactions impact HIV-1 infection, transmission, and neutralization. Notably, the interactions of HIV-1 with *O*-glycan-binding SLBR lectins that are integral parts of the bacteria fimbriae from commensal oral *Streptococcal gordonii* strains enhanced infectivity of cell-free virions. SLBR-N, one of the SLBRs which recognizes sialyl Lewis X (sLe^X^), displayed the greatest activity, although the activity varied among different HIV-1 strains, reflecting glycan variability among Env strains. SLBR-N also promoted transmission of cell-associated virus to CD4 T cells. In addition to the SLBR lectins, *O*-glycan-specific plant lectins increased HIV-1 infectivity, indicating a specific effect of *O*-glycan engagement. The mechanisms by which SLBR-N and other *O*-glycan-binding lectins increase HIV-1 infectivity are not fully understood, although our data indicate that SLBR-N-mediated enhancement may occur at an early step during the initial virus-target cell interactions prior to virus fusion. Our study further implies a potential role for bacteria that colonize the host mucosa surfaces in influencing HIV-1 infectivity and determining the risk of HIV-1 transmission, even though the in vivo significance of these findings requires more investigation.

Although core 1 and/or core 3 *O*-glycans have been reported on gp120 from HIV-1, HIV-2, and SIV, the role of *O*-glycans in HIV-1 biology and pathogenesis has been largely understudied. We used a microarray technology with lectins of distinct specificities to identify the enrichment of *O*-glycans and glycans with terminal sialic acids or sulfates as general signatures of HIV-1 virions across different strains. Our experiments with the SLBR lectins further indicated the presence of *O*-glycans with α−2,3-sialic acid and potentially sLe^X^ detected by SLBR-N on the surface of HIV-1 virions and on virus Env glycoproteins. Of note, CD4+ T cells with active HIV-1 replication were found to display higher cell-surface levels of sLe^X^ compared to cells with transcriptionally inactive infection^54^. CD4 T cells with higher sLe^X^ levels also expressed markers associated with HIV-1 susceptibility, as well as intracellular signals known to promote HIV-1 transcription and associate with leukocyte extravasation^54^. It also has been shown that HIV-1 produced in cells deficient of *O*-glycosylation were more sensitive to bNAbs targeting the V3 glycans, indicating the involvement of *O*-glycans in shielding the V3 glycan epitopes. Our study further showed that the SLBR-N binding to *O*-glycans on Env rendered HIV-1 more resistant to bNAbs against the CD4bs and the V1V2 glycan. While such bNAbs are produced only by a subset of HIV-1 infected individuals^55–57^ and the impact of bNAb epitope shielding on virus escape during natural infection might not be widespread, the ability of *O*-glycans and *O*-glycan-binding lectins to modulate bNAb potency may be factors to consider when bNAbs are utilized as prophylactic and therapeutic agents. Hence, we have demonstrated here that lectins from bacteria in the human mucosal microbiota can interact with sialyated *O*-glycans on the virus surface and that *O*-glycan recognition specifically affect virus infection, cell-cell transfer, and neutralization by antibodies, signifying a distinctive contribution of *O*-glycans to HIV-1 pathogenesis.

Along with *O*-glycans, high mannose-type *N*-glycans were found to be enriched in HIV-1 virions in line with past reports^38–40^. However, high mannose specific lectins FimH from *E. coli* and Msl from *L. plantarum*, which were studied in tandem with the SLBRs, had little effect on virus infectivity, even though they similarly captured viral particles and recognized HIV-1 Env. On the other hand, high-mannose-specific plant or algae lectins are known to have antiviral activity against HIV-1, including GRFT which is under development for antiviral microbicides ^41, 58^. One possible reason for the functional differences between *O*-glycan and *N*-glycan engagement is that, unlike the high density of *N*-glycans on HIV-1 Env, fewer *O*-glycosylation sites are predicted on each Env subunit and they are localized in discrete regions, particularly the highly variable loops and at a conserved C5 site near the furin-cleavage site (**Supplemental Figure 2**), lessening the likelihood for multivalent interactions. Indeed, GRFT is a dimer with multiple putative binding sites per subunit and its antiviral potency has been associated with multivalent interactions with HIV-1 Env ^58, 59^, whereas non-inhibitory FimH and Msl lectins are monomers with one glycan recognition site. In the case of *O*-glycan-binding lectins, monomeric SLBR-N with a single glycan-binding site^60^ and multimeric Jacalin (AIA)^61^ and MAL II^62^ showed the ability of enhance HIV-1 infection. Enhanced infection also was similarly observed with SLBR-N with or without GST, a tag protein that forms homodimers (**Supplemental Figure 3B**), indicating that multivalency may not be requisite for this effect, although the self-association of SLBR-N made as recombinant soluble protein cannot be ruled out.

We also found that lectin treatment of gp120 had no effect on CD4 binding to gp120. Rather, we postulate that lectins binding to sialic acid moieties on *O*-glycan structures may mask these negative charges on the virion surface and promote the interactions between virions and target cells. Indeed, semen-derived enhancer of viral infection (SEVI), which is an amyloid fibril found in semen made of cationic peptide fragments from prostatic acidic phosphatase, has been shown to increase HIV-1 infectivity by capturing HIV-1 virions and augmenting attachment to target cells^63^. The polycationic nature of SEVI was found to neutralize the negative charge repulsion between HIV-1 virions and target cells therefore promoting infection^64^. Polymers such as polybrene and DEAE Dextran that are commonly used to facilitate HIV-1 infection and lentivirus transduction also enhance virus-cell binding through a similar mechanism^65^. Apart from viral Env, *O*-glycan-bearing cell membrane proteins such as CD162 (P-selectin glycoprotein ligand-1), CD43 (sialophorin), and CD44 (E-selectin ligand), are present on the surfaces of HIV-1 virions^66–68^. Nonetheless, the extent to which SLBR-N and similar lectins bind to *O*-glycans on cellular proteins relative to Env is yet unknown. The capacity of these lectins to augment virus attachment to cells also requires further examination.

We observed that Jacalin and MAL II enhanced HIV-1 infection, albeit with a distinct dose response from that of SLBR-N. Jacalin and MAL II, but not SLBR-N, also enhanced HIV-1 infection upon interacting with cells after virus infection. In this case, crosslinking of *O*-glycan-bearing ligands on the cell surface by these multimeric lectins may impart intracellular signal activation to affect cell metabolism and virus replication. Of note, in a report by Silver et al.^18^ Jacalin treatment was shown to inhibit infection of recombinant chimeric NL4–3 viruses, but potent inhibition was seen only for one HIV-1 strain whereas other strains required relatively high lectin concentrations of 250–1000 μg/mL for inhibition, and for one strain enhancement of infection was apparent. In this study, we tested Jacalin from 0.01 to 50 μg/mL and observed enhancement of infection that peaked at 10 μg/mL and declined at higher concentrations; this pattern was seen with full length IMCs of acute or transmitted/founder HIV-1 strains from different subtypes and with viruses produced in HEK293T or PBMCs. However, we have tested only few IMC strains thus far and Jacalin displayed varying levels of enhancement for the different IMCs, offering the possibility for wide-ranging effects on the highly heterogeneous strains and isolates of HIV-1.

This study revealed that SLBR lectins from oral Streptococcal bacteria can bind HIV-1 virions and augment virus infectivity. Streptococci including SLBR-expressing *S. gordonii* and closely related *S. mitis* strains are among the most common genera that colonize the human oral cavity, the upper gastrointestinal tract and the genitourinary tracts, and are abundantly present in milk^69–74^. Reminiscent of findings observed for a number of enteric viruses ^2–9^ and MMTV ^10, 11^, direct interactions between HIV-1 and bacteria or bacterial products in the host microbiota may constitute a critical determinant for HIV-1 acquisition through mucosal routes, including mother to child transmission during perinatal and breastfeeding periods and sexual intercourse activities involving oral and urogenital contacts. The study reported here is also pertinent and timely given that HIV-1 glycans are the main targets for antiviral lectins being explored as candidate microbicides^41, 58^ and for many bNAbs under development for HIV-1 prophylactics^75, 76^.

### Limitations of the study

The present study is limited to in vitro experiments with recombinant purified lectins. HIV-1 interactions with SLBR lectins as expressed on bacterial fimbriae and their in vivo consequences have not yet been evaluated. The amounts of bacterial lectins at the mucosal sites are also unknown. However, considering that 10^7^ Streptococci have been detected in 10 uL of saliva and a much higher number is expected in a biofilm on dental surface and oral mucous membrane^77, 78^ and that each bacterial cell has hundreds of lectin-capped fimbriae, the lectin concentration in such a micro-environment may be relatively high, reaching the concentration range tested in this study. For a 50 kD SLBR-N, 0.3 to 300 μg/mL is equivalent to 3.6 × 10^9^ to 3.6 × 10^12^ molecules/μL. The study also did not examine the effect of these lectins on virus uptake by and transinfection from dendritic cells and other myeloid cells which participate in the initial stages of virus acquisition at the mucosal tissues^23,24, 25,26^. Nonetheless, our findings provide an impetus for investigations into HIV-1 interactions with the host microbiota that are requisite for formulating more effective modalities to prevent HIV-1 infection.

## STAR Methods

### RESOURCE AVAILABILITY

- Lead Contact: Further information and requests for resources and reagents should be directed to and will be fulfilled by the lead contact, Catarina E. Hioe (catarina.hioe@mssm.edu, catarina.hioe@va.gov).
- Materials Availability: This study did not generate new unique reagents.
- Data and Code Availability:
  - All data reported in this paper will be shared by the lead contact upon request.
  - This paper does not report original code.
  - Any additional information required to reanalyze the data reported in this paper is available from the lead contact upon request.

### EXPERIMENTAL MODEL AND SUBJECT DETAILS

#### Cell lines, lectins, anti-Env mAbs, and viruses

TZM-bl cell line was obtained through the NIH AIDS Research and Reference Reagent Program (ARRRP), contributed by J. Kappes and X. Wu. HEK293T cells were obtained from the American Type Culture Collection (ATCC). The TZM-bl cell line was maintained in Dulbecco’s modified eagle medium (DMEM; Lonza) supplements with 10% heat-inactivated FBS, gentamicin (50 μg/mL), and HEPES (25mM) and the HEK293T cell line was maintained in DMEM containing 10% heat inactivated fetal bovine serum, penicillin/streptomycin (100U/mL), and L-glutamine. Jurkat T cells (E6–1) from Dr. Arthur Weiss (ATCC) were obtained from the NIH HIV Reagent Program (HRP) and maintained in complete RPMI medium (RPMI 1640 medium with 10% FBS, 100U/mL penicillin, 100μg/mL streptomycin, and 2mM glutamine). Primary CD4+ T cells were obtained from isolation of human peripheral blood through the New York Blood Center. Isolation was performed with R&D Systems MagCellect Human CD4+ T cell Isolation Kit (Fisher Scientific). Primary CD4+ T cells were maintained in complete RPMI medium.

The recombinant FimH protein, which contains only the lectin domain, was produced in *E. coli* as described in^79^. SLBRs^60^ and Msl^34^ were similarly produced in *E. coli* transformed with the respective plasmids. All plant lectins were purchased from Vector Labs.

All anti-Env mAbs (NIH45–46^80^, PG9^81, 82^, 2219^83, 84^) were generated by transfecting plasmid DNA into 293F cells and collecting supernatants after 5–7 days of culture. MAbs were purified using a Hitrap protein A column (GE Healthcare).

The following reagents were obtained through the NIH AIDS Reagent Program, Division of AIDS, NIAID, NIH: HIV-1 Z331M T/F Infectious Molecular Clone from Dr. Eric Hunter and pREJO.c/2864, contributed by Dr. John Kappes and Dr. Christina Ochsenbauer. Recombinant IMCs of B.JRFL^83, 85^ and AE.CMU06^40^ were obtained from Dr. Jerome A. Zack (UCLA) and Dr. Chitra Upadhyay (Icahn School of Medicine at Mount Sinai), respectively. These IMCs were generated by transfection of HEK293T using jetPRIME^®^ (Polyplus). Supernatants were filtered (0.45 micron) and pelleted through a 20% sucrose cushion by ultracentrifugation. Viral pellets were resuspended in PBS, tittered on TZM-bl cells, aliquoted, and stored at −80°C until use.

4102–61 is a replication competent HIV-1 clone produced in transfected 293T cells and passaged on primary CD4 T cells^86^. NLCI is an X4-tropic HIV-1 IMC expressing NL4.3 Env and an mCherry reporter in the *nef* locus with Nef expression restored by the use of an internal ribosome entry site (IRES)^87^. SHIV stocks were produced in 293T cells^88–90^. The transmitted/founder (T/F) HIV-1 QH0692 Env (HRP Cat #11227, Drs. David Montefiori and Beatrice Hahn) was cloned into pNL4–3 based HIV-1 Gag-iCherry backbone as previously described^91^. To generate a cleavage-defective Env mutant^92^, we introduced mutations at the primary cleavage site R519S and R522S by PCR amplification using CloneAMP (Takarabio). Mutated PCR fragment was inserted into pNL4–3-QH0692 plasmid with EcoRI-HF and MIuI-HF, with the In-Fusion HD Cloning Kit (Fisher Scientific). HIV-1 Gag-iCherry was digested with EcoRI-HF and XhoI-HF, followed by Gibson assembly (New England BioLabs). The mutants were confirmed by Sanger sequencing across all amplified regions.

### METHOD DETAILS

#### Lectin Microarray

Lysates were produced from viruses and the cells from which the viruses by treatment with Triton X-100 (1%) in PBS. After centrifugation, lysates were analyzed for protein concentrations using a DC Assay (BioRad). Each lysate sample (10 μg of protein) was labeled with Alexa Fluor 555-NHS. A reference sample created by pooling equal protein amounts from each lysate was labeled with Alexa Fluor 647-NHS. Printing and hybridization were performed as previously described^36, 93, 94^. The print list for our lectin microarray is shown in **Supplemental Table 1**. Log_2_ values of the average signals for each lectin are median-normalized over the individual subarray in each channel. Hierarchical clustering using the Pearson Correlation Coefficient, heatmap generation, and data analysis was performed using R (version 1.3.109). Relevant glycan structures were determined using the known specificities for each lectin^95^. Glycan changes were highlighted if they were consistent for all viral versus cell lysates, were observed for lectins that share similar binding motifs and their specificity was unambiguous, and at least one of the lectins was statistically significant (as determined by Student *t* test).

#### Virus infection assay

Virus was mixed with titrated amounts of each lectin and incubated at 37°C for 1 hr or up to 24 hrs for some experiments. TZM-bl cells were then added (5,000 cells per well with DEAE dextran at 30 μg/mL) and incubated for 48 hrs. Virus infection was detected using britelite^™^ Plus reagent (PerkinElmer) and luminescence was measured using a BioTek Cytation^™^ 3 luminometer. Virus input was pre-determined to produce RLU values between 100,000 and 200,000. RLUs were normalized to untreated controls (set to 100%). Cells were separately treated with lectin and viability was measured using PrestoBlue Cell Viability Reagent (ThermoFisher). Each condition was tested in triplicate.

For some experiment, virus was pre-treated with O-glycoprotease (20 μl, 1000 units/mL, New England Biolabs) or with neuraminidases from *Clostridium perfringens* (C.p) (2 μl, 5 units/mL, Sigma) or *Arthrobacter ureafaciens* (A.u) (1 μl, 10 units/mL, Roche) or both^42^. In a separate experiment, AIA was pretreated with melibiose (Sigma-Aldrich) for 1 hr at 37°C. Melibiose was maintained at a final concentration of 100 mM throughout the assay.

For neutralization assays, B.JRFL or C.Z331M were treated with bNAbs NIH45–46 (1 μg/mL) or PG9 (10 μg/mL) for 1 hr at 37°C, and then incubated with titrated amounts of SLBR-N (1:5 dilution starting at 200 μg/mL) for 1 hr at 37°C. This mixture was then added to TZM.bl reporter cells for 48 hrs. Similar experiments were performed where B.JRFL was sequentially treated with SLBR-N (200 μg/mL) for 1 hr at 37°C and titrated amounts of mAbs 2219 (1:5 dilution starting at 50 μg/mL) for 1 hr at 37°C, then mixed with TZM.bl reporter cells for 48 hrs.

#### Virus Capture

Virus was incubated with SLBR-N (20 μg) or left untreated for 24 hrs at 37°C. Pierce^™^ Glutathione Magnetic Agarose (100 μl, ThermoFisher) was added to the mixture and incubated for 1 hr at 37°C and then pelleted. The supernatant was titrated on TZM.bl cells to measure residual virus infectivity. The beads were washed 3 times with PBS to remove unbound virus and subjected to vRNA quantification by real time PCR using the Abbott m2000 System^83^. The beads were also titrated on TZM.bl cells to measure virus infectivity of captured viral particles.

#### Lectin Blotting

Recombinant HIV-1 Env were resolved on a 4–20% gradient SDS Page gel (Bio-Rad) and transferred to a nitrocellulose membrane using an iBlot 2 transfer device. The membrane was blocked using either SuperBlock^™^ (PBS) Blocking solution (ThermoFisher) or Blocker^™^ BSA 10% in PBS (ThermoFisher). FimH (2 μg/mL) or SLBR-N (1 μg/mL) was then added in blocking buffer and incubated for 1 hr at room temperature. Blots were washed with PBS-T (PBS with 0.05% Tween-20, pH 7.4, 3x, 5 min each) and THE^™^ His Tag mouse antibody (Genscript, 0.5 μg/mL) for FimH or anti-GST rabbit antibody (Abcam, 1 μg/mL) for SLBR-N was added in blocking buffer and incubated for 1 hr at room temperature. Blots were washed again in PBS-T (3x, 5min) and incubated with anti-Mouse antibody HRP or anti-Rabbit antibody HRP (1:1000, KPL Antibodies and Conjugates) for 1 hr at room temperature. After final wash, the blots were developed using the ECL substrate (BioRad) and luminescence was detected using a BioRad ChemiDoc^™^ MP imaging system.

To detect Env bands, blots were stripped using Restore^™^ PLUS Western Blot Stripping Buffer (ThermoFisher) to remove lectins, blocked with 5% milk in PBS-T, and probed with a pool of monoclonal human antibodies against HIV-1 Env. Env bands were detected using an anti-Human Ig HRP antibody (1:1000, KPL Antibodies and Conjugates) and visualized as described above.

For some blots, Env (1–5 μg) was pretreated with O-Glycoprotease (1 μl), α2–3,6,8 neuraminidase/O-glycosidase (2 μl each), EndoH (5 μl), or PNGaseF (1 μl) for at least 1 hr at 37°C using denaturing conditions. All enzymes were purchased from New England Biolabs and the manufacturers protocol was used.

#### Biolayer interferometry

Binding kinetics of bacterial lectins for HIV Env were performed by biolayer interferometry using an Octet Red96 instrument (ForteBio/Sartorius). Recombinant gp120 A244 or JRFL (10 μg/mL) were coupled to Octet^®^ AR2G Biosensors (Sartorius) following the manufacturers protocol. Biosensors were then dipped into titrated amounts of SLBR-N. For FimH, anti-Env monoclonal antibody 2219^83, 84^ (5 μg/mL) was immobilized on Octet^®^ AHC Biosensors (Sartorius) followed by recombinant gp120 ZM109 (5 μg/mL). Biosensors were then dipped into titrated amounts of FimH. These experiment measured the affinity of each lectin for gp120 glycans in a 1:1 stoichiometry. Samples were diluted in PBS supplemented with BSA (0.1% w/v) and Tween 20 (0.02% v/v). A loaded sensor run with a buffer blank was used as reference to correct for drift. Reference curves were subtracted, and the data was analyzed with the Octet Data Analysis software by employing a 1:1 homogenous binding model for a global fit analysis for association and dissociation curves.

#### ELISA

To measure SLBR-N binding to gp120, recombinant versions of HIV-1 Env (C.1086gp140K160N, gp120 ZM109, and gp120 JRFL) were coated on the well surface overnight at 4°C. The wells were washed with PBS-T (PBS with 0.05% Tween-20, pH 7.4, 3x) and then blocked with 3% BSA in PBS for 1 hr at 37°C. The wells were then washed again and titrated amounts of GST-tagged SLBR-N (1:3 starting at 40 μg/mL) were incubated for 1 hr at 37°C. Wells were subsequently washed and an anti-GST rabbit antibody (Abcam, 1 μg/mL) was added for 1 hr at 37°C, wells were washed, and SLBR-N binding was measured using anti-Rabbit HRP (1:1000, KPL Antibodies and Conjugates, 1 hr at 37°C). After the final washes, the ELISA was developed by measuring luminescence using the SuperSignal^®^ ELISA Pico Luminol/Enhancer Solution.

To measure the impact bacterial lectins have on CD4 binding to gp140, recombinant C.1086gp140K160N was coated on the well surface overnight at 4°C. The wells were washed with PBS-T (PBS with 0.05% Tween-20, pH 7.4, 3x) and then blocked with 3% BSA in PBS for 1 hr at 37°C. The wells were then washed and incubated with bacterial lectins (FimH and SLBR-N, 50 μg/mL) or PBS. The wells were washed and incubated with titrated amounts of soluble CD4 (1:3 from 10 μg/mL, NIH AIDS Reagent Program, Division of AIDS, NIAID ARP-4615, Progenics) for 1 hr at 37°C. After washing, anti-Human CD4 Biotin (OKT4, Invitrogen, 1 μg/mL) was added for 1 hr at 37°C, followed by washing, and subsequent detection using Streptavidin AP (Invitrogen, 1:1000). The wells were washed a final time and luminescence was measured using KPL PhosphaGLO^™^ AP substrate (SeraCare Life Sciences).

#### Cell-to-cell transfer assay

Jurkat T cells were nucleofected with HIV-1 Gag-iCherry using Cell Line Nucleofector Kit V (Lonza) and incubated overnight at 37°C. After 16–18 hours, live nucleofected Jurkat (donor) cells were isolated via Ficol density gradient centrifugation. Donor and target primary CD4+ T cells were dye-labeled with Cell Proliferation Dye eFluor 670 and eFluor 450 respectively (Invitrogen). 1×10^5^ donor cells were co-cultured with equal number of target cells in a round bottom 96-well plate. After 3 hours, cells were washed and trypsinized to remove surface-attached virus particles and trypsin activity was neutralized with complete RPMI media. Cells were then washed and fixed with 2% paraformaldehyde (PFA) for 20 minutes at room temperature, then run on Attune Flow Cytometer (ThermoFisher). Positive mCherry signal from transferred HIV Gag-iCherry virus particles from eFluor 450+ cell population represents internalized virus particles transferred from cell to target cell. Neutralization of cell-to-cell transfer assay was performed by preincubating lectins or anti-CD4 antibody, Leu3a, with donor cells for 30 minutes at 37°C prior to cell mixing. Leu3a was also preincubated with acceptor cells.

### QUANTIFICATION AND STATISTICAL ANALYSIS

Virus infectivity data were calculated and plotted using untreated virus controls set to 100%. Statistical analysis was performed in GraphPad Prism using statistical tests designated in the figure legends. Hierarchical clustering using the Pearson Correlation Coefficient, heatmap generation, and data analysis was performed using R (version 1.3.109).
